# Evaluation of misinformation among pro-Ukrainian Latvians – the role of prior attitude, analytical thinking, and emotions

**DOI:** 10.3389/fpsyg.2023.1165039

**Published:** 2023-09-14

**Authors:** Martins Priedols, Girts Dimdins

**Affiliations:** Department of Psychology, University of Latvia, Riga, Latvia

**Keywords:** analytical thinking, emotions, misinformation, motivated reasoning, myside bias

## Abstract

In this exploratory study with a community sample (*N* = 115), we look at the perception of pro-Russia and pro-Ukraine misinformation, mimicking content shared by naive Facebook users, and the factors related to it among pro-Ukraine Latvians. Our results support the integrative model in the perception of misinformation—we found strong evidence of myside bias, as pro-Russia misinformation was judged to be significantly less accurate than pro-Ukraine misinformation. Analytical thinking, measured with the seven-item cognitive reflection test, was associated with lower levels of pro-Ukraine misinformation accuracy judgments and lower overall misinformation accuracy judgments; however, there was no correlation between analytical thinking and pro-Russian misinformation accuracy judgments. Pro-Ukrainian misinformation accuracy judgments were positively related to positive emotions elicited by misinformation, the level of support for Ukraine, and the participant's age. In addition, participants indicated a higher likelihood of engaging with misinformation if they came across it online, trusted the information, and if it elicited positive emotions. Thus, our findings emphasize the role of one's attitude, analytical thinking, and emotions in one's perception, evaluation, and engagement with congruent and incongruent misinformation.

## 1. Introduction

Misinformation (false information that is spread, regardless of intent to mislead), including disinformation (deliberately misleading or biased information spread with the intent to mislead), “fake news,” propaganda, and conspiracy theories, present significant social, economic, and political problems worldwide (Van Bavel et al., 2021). For some time, the rise of such online content has also been noticed in Central and Eastern Europe regarding such topics as COVID-19, politics, and, more recently, the war in Ukraine (Faragó et al., 2023). In the context of Russian aggression, widespread disinformation campaigns and user-created misinformation are a threat not only to established democratic systems through the rise of illiberal populism but also to national security. Indeed, online influence operations are a significant concern due to low barriers to entry and scalability (Alizadeh et al., 2020). While the disinformation campaigns and perception of Russia's and its allies' propaganda have been extensively studied (Morkunas, 2022), disinformation in war is a vital part of the playbook for all sides, and some of Ukraine's official accounts have endorsed information that was later proved false (Thompson and Alba, 2022). In addition, misinformation can be spread unintentionally, such as when information from the battlefield is effectively shared in real-time before it can be checked and its sources verified. In such conditions, the audience's ability to recognize misinformation is of vital importance. A key goal of this study is to explore the relationships between individuals' prior beliefs, analytical thinking, and emotional perception when evaluating misinformation's accuracy and (in)congruent content, using the war in Ukraine as a background of current importance.

Despite its large Russian population, Latvian society has strongly supported Ukraine in the war against Russia. Partly, this is due to historical preconditions—Latvia formed part of the Soviet Union and was among the primary targets for Russian disinformation (Morkunas, 2022). In Russia, the Baltic countries, including Latvia, are referred to as “near abroad”—a unique term coined in 1992 that tries to highlight their status as foreign countries not fully recognized by Russia (Rotaru, 2017). Therefore, the war in Ukraine has overwhelmed the nation with various feelings—on the one hand, empathy for the suffering and bravery of Ukraine; on the other, fear of Russia aiming to conduct similar actions in the Baltic states.

In a national survey (Latvijas Fakti, 2022), an overwhelming majority of Latvian society showed its support for Ukraine in the fight against Russian aggression, for the reception of Ukrainian refugees, and for Ukraine's admission to NATO. Ukraine's struggle for independence and freedom of its land was supported by 82% of surveyed Latvian residents, 73% supported the reception of Ukrainian war refugees in Latvia, and 63% supported Ukraine's admission to NATO. On the other hand, only 4% of respondents supported the Russian invasion of Ukraine. In addition, Latvian citizens highly valued their media literacy skills—the majority of participants (57%) believed they could distinguish true information about the war in Ukraine from distorted information. Combined with the historical facts, the circulation of pro-Ukraine and pro-Russia misinformation that has been a part of the Latvian media landscape has increased since the Russian invasion. Though most of the information comes from trusted sources, such as the Public Broadcasting of Latvia, the amount of questionable content, opinions, and personal accounts of users has surged. Therefore, misinformation that users besides public media outlets produce is a part of everyday misinformation, and this is the focus of our investigation.

Two main models of reasoning are usually used to describe why people believe in misinformation. The first is “motivated system 2 reasoning” (Kahan, 2013), where the ability to think analytically increases partisan-motivated reasoning. Kahan's findings identify ideologically motivated cognition that encourages individuals to form and maintain subjectively important beliefs—people with a higher level of analytical thinking are expected to believe in ideologically consistent misinformation and use their mental capacity to strengthen their existing beliefs (Kahan, 2013; Drummond and Fischhoff, 2017). Kunda (1990) approach emphasizes motivation as an integral part of reasoning by relying on a biased set of cognitive processes, including strategies for accessing, constructing, and evaluating beliefs. According to Ziva Kunda's concept of motivated reasoning, people are motivated to reach conclusions that they want to reach. While both theories agree that incentives can guide reasoning, the analytic thinking model proposes that people employ their cognitive resources consciously to reinforce their prior views. The primary distinction is between one model's active involvement in analytic processes and Kunda's broader, more generalized, motivation-driven reasoning.

The second model is the “classical reasoning theory”, which suggests the opposite mechanism where not engaging in analytical thinking is the main reason behind belief in misinformation. According to this approach, analytical thinking (measured by the cognitive reflection test [CRT]) decreases the perceived accuracy of misinformation regardless of its consistency with prior beliefs (Pennycook and Rand, 2019a,b; Pehlivanoglu et al., 2021; Faragó et al., 2023) and also reduces engagement with false content (Ross et al., 2021).

Indeed, research supports this approach, as analytical reasoning has sometimes been found to override default responses suggested by intuitive processes (Evans and Stanovich, 2013). Through this process, employing analytical versus intuitive reasoning may reduce gullibility, decreasing support for misinformation (Bronstein et al., 2019).

In the context of the war in Ukraine, both models of reasoning have been investigated within the scope of belief in pro-Kremlin disinformation among pro-Russia Ukrainians (Erlich et al., 2022). Authors find support for the classical reasoning model when analytical thinking is associated with a better ability to distinguish truth from disinformation among Ukrainians who are strongly oriented toward Russia. If taken to an extreme, this model suggests that the analytical thinking described by the CRT is a more robust predictor of susceptibility to misinformation than individuals' ideological beliefs (Roozenbeek et al., 2022).

In addition to the classical and motivated reasoning theories, the integrative model, which emphasizes the role of individuals' ideology through political partisanship or myside bias, should be noted (Van Bavel et al., 2021; Roozenbeek et al., 2022; Van der Linden, 2022). Myside bias occurs when people assess evidence, produce evidence, and test hypotheses in a way that is biased toward their attitudes and prior beliefs (Stanovich et al., 2013). The level of myside bias shows minimal relation to intelligence, and avoiding it is an analytical thinking skill that cannot be assessed or indexed by such cognitive ability measures as CRTs. Thus, partisanship can be a stronger predictor of belief in congruent misinformation and disbelief in incongruent misinformation than analytical thinking (Van der Linden, 2022). Although cognition and analytical thinking skills may positively influence one's ability to identify misinformation, partisanship, and identity-related motivations better explain one's susceptibility to misinformation (Roozenbeek et al., 2022). The fundamental difference between the integrative model and classical and motivated reasoning theories is seen in motivation. Motivated thinking is deliberately motivated by a specific desire or emotion, and it uses cognitive resources to reach a desired conclusion, even if it requires an additional mental load. Myside bias, on the other hand, might be considered a broad bias toward one's point of view, sometimes without a specific emotional or intended purpose driving the reasoning process.

Recently, Martel et al. (2020) observed that disinformation authors use very emotional content that is processed quickly and can cause difficulty in recognizing what is true. Negative emotions, such as anger, fear, and disgust, have previously been noted as pathways toward superficial engagement and, therefore, one's susceptibility to disinformation. Usually, something positive will have less of an effect on one's behavior and perception than something equally intense yet negative (Baumeister et al., 2001).

However, previous studies have looked at misinformation in a somewhat calmer context and, usually, by addressing misinformation in opposition to general knowledge. Emotions can be flexible in influencing cognition—both positive and negative emotions have been shown to influence judgment and depend on the nature of the task and topic (Huntsinger and Ray, 2016). In the case of war, it has been noted that the misinformation used by Ukrainian sources and oriented toward Ukrainians focused mainly on its heroes and sacrifices, characters who help dramatize tales of Ukrainian endurance and Russian violence, serving as morale boosters (Thompson and Alba, 2022). Using “positive” propaganda is nothing new. For example, during World War I, the sole purpose of some British journalists was to spread positive messages regarding Britain, thereby countering the propaganda of enemy countries (Jain, 2006). Thus, positive emotions could also be the driver of belief in misinformation if they support one's beliefs and allegiance.

Drawing on previous findings, we tested several predictions regarding whether respondents would evaluate misinformation differently if it was congruent or incongruent with their existing beliefs, i.e., support for Ukraine, and if their existing position is associated with the confirmatory evaluation of information as suggested by the integrative model. Therefore, our first prediction (P1) is*: “Pro-Ukraine participants will rate pro-Ukraine misinformation as more accurate than pro-Russian misinformation.”*

Second, analytical thinking, as described by the CRT, should significantly influence one's belief in both congruent and incongruent misinformation and the likelihood of engagement. This would be consistent with the classical reasoning theory; therefore, “*Higher levels of analytical thinking (CRT results) will be negatively related to misinformation accuracy judgment in both pro-Ukraine and pro-Russia misinformation”* (P2) and “*Higher levels of analytical thinking (CRT results) will be negatively related to the likelihood of engagement with pro-Ukraine and pro-Russia misinformation”* (P3).

Misinformation that generates powerful emotional responses, such as fear, wrath, or delight, is more likely to be believed and spread (Brady et al., 2017). A piece of information's emotional impact can eclipse its factual truth. As previous research (e.g., Baumeister et al., 2001; Huntsinger and Ray, 2016) on the relationship between elicited emotions and the evaluation of (mis)information has shown mixed results, we also included emotional intensity measures to see if they are related to the perception of congruent and incongruent misinformation in an exploratory manner.

## 2. Participants

One hundred fifteen respondents aged between 18 and 75 (82.6% female, M = 32.69, SD = 13.39) participated in the survey. We used the QuestionPro platform to collect data from the online survey and distributed the survey to multiple public discussion boards on Facebook in October 2022. We planned to collect answers from a minimum of 108 and a maximum of 200 respondents by 15 October 2022 (whichever came first). *A priori* sample power analysis with a statistical power of 0.8 and an anticipated medium effect size determined the minimum sample size of 108 participants. Participation in this study was voluntary, anonymous, and without any remuneration.

## 3. Materials and procedure

Following a short demographic questionnaire (age, gender, and level of education), all participants answered two questions about their attitude toward the ongoing war in Ukraine on a 7-point Likert scale, ranging from 1 (“I completely disagree”) to 7 (“I completely agree”). Questions included “I support Ukraine in the ongoing hostilities” and “I support Russia in the ongoing hostilities”. Though these questions were initially meant to create an index of support for Ukraine, internal validity analysis suggested that these two measures were not sufficiently aligned to be combined into a single index (Cronbach's α = 0.33). Therefore, they were included in the analysis separately.

After the initial questionnaire, the participants read the following introduction: “In the following, you will see screenshots from the social network “Facebook”. Each of them will be followed by questions about your opinion and emotions in the event you came across such a post on Facebook.”

The participants continued with the evaluation of eight stimuli (four pro-Ukraine and four pro-Russia), where information was presented as screenshots of Facebook user posts. The author, comments, likes, and shares were redacted. The post content included a variety of misinformation, both pro-Russia (e.g., “The latest data show that up to 80% of the weapons transferred to Ukraine cannot be used because there are not enough human resources! So why are Ukrainians still asking for weapons? To sell on the black market?”) as well as pro-Ukraine (e.g., “For some reason, the number of broken legs among men who could be drafted into the army has recently increased ten-fold in St. Petersburg… For some reason”). All the stimuli were created for this research and reviewed by authors and independent misinformation experts to contain information that participants might plausibly believe and resemble contemporary misinformation. At the time of data collection, all stimuli contained some form of misleading or factually incorrect information and thus could be counted as misinformation.

Posts were shown to participants in a random order, and after viewing the posts, participants answered two questions about their truthfulness (“This post seems credible” and “This post contains misleading or false information”) on a 5-point Likert scale, ranging from 1 (“I completely disagree”) to 5 (“I completely agree”). After reversing the second question, two indexes were calculated—one for pro-Ukraine misinformation accuracy judgment (truthfulness index) and the other for pro-Russia misinformation accuracy judgment (truthfulness index)—by averaging the accuracy ratings of pro-Ukraine stimuli and pro-Russia stimuli, respectively. The two indices had adequate internal consistency (Cronbach's α = 0.69 and 0.75, respectively).

In addition, each post was rated based on the emotional response elicited by the misinformation. Respondents were asked to rate emotions in response to the question, “To what extent does this record make you feel ___?” on a 5-point Likert scale, ranging from 1 (“Not at all”) to 5 (“Very”). The emotions they were asked to evaluate were “Happiness”, “Enthusiasm”, “Pride”, “Anger”, “Fear”, “Sadness”, and “Disgust” (Marcus et al., 2006). The mean scores of positive (“Happiness”, “Enthusiasm”, “Pride”) and negative (“Anger”, “Fear”, “Sadness”, and “Disgust”) emotions were calculated, and separate indexes for emotions elicited by pro-Ukraine and pro-Russia content as well as the overall indexes were created. All indexes—positive emotions elicited by all misinformation (Cronbach's α = 0.64), pro-Ukraine misinformation (Cronbach's α = 0.58), and pro-Russia misinformation (Cronbach's α = 0.69), and negative emotions elicited by all misinformation (Cronbach's α = 0.92), pro-Ukraine misinformation (Cronbach's α = 0.87), and pro-Russia misinformation (Cronbach's α = 0.88)—had acceptable internal validity.

The likelihood of engagement with the misinformation was calculated by indexing responses to determine whether the participant would be likely to share and comment on the particular content. Participants rated this likelihood on a 5-point Likert scale ranging from 1 (“Not at all likely”) to 5 (“Very likely”) in response to two questions: “What is the likelihood that you would share this post?” and “What is the likelihood that you would comment on this post?” The overall “Likelihood of engagement” index had acceptable internal validity (Cronbach's α = 0.90).

At the end of the survey, after evaluating the eight Facebook posts, respondents were given the 7-item CRT (Toplak et al., 2013) (e.g., “A farmer had 15 sheep, and all but 8 died. How many are left?” The intuitive answer is 7; the correct answer is 8). After responses were coded as right (1) or wrong (0), the mean was calculated (the higher the mean, the more analytically a person thinks). The scale had adequate internal consistency (Cronbach's α = 0.66).

After completing the survey, participants were debriefed and informed that all the information used in the research was misinformation that contained false facts and interpretations of events and was created for the research rather than being an actual representation of Facebook user posts.

## 4. Results

Descriptive statistics of the responses to attitudes toward the ongoing war in Ukraine showed high support for Ukraine (*M* = 6.42, *SD* = 1.26) and low support for Russia (*M* = 1.17, *SD* = 0.74). A total of 74.8% of all respondents indicated that they strongly support Ukraine, while only 5.2% leaned toward disagreeing with this question. When asked about their support for Russia, 92.2% of participants indicated that they strongly disagreed with this position; only one participant (0.9%) strongly supported Russia.

A repeated measures ANOVA was performed to compare misinformation accuracy judgment ratings between pro-Ukraine and pro-Russia stimuli. There was a statistically significant difference in misinformation accuracy judgment ratings between the two groups [*F*_(1, 114)_ = 210.682, *p* < 0.000, η^2^ = 0.649], and the mean value of misinformation accuracy judgment was significantly different between the pro-Ukraine misinformation ratings (*M* = 3.05, *SD* = 0.63) and pro-Russia misinformation ratings (*M* = 1.99, *SD* = 0.61), thus confirming our first prediction. To test the relationship between prior attitude and analytical thinking on the misinformation accuracy judgment ratings, we added support for Ukraine, support for Russia, and CRT results to the analysis as covariates. The result showed a significant interaction between misinformation accuracy judgment ratings and the level of support for Ukraine [*F*_(1, 111)_ = 16.585, *p* < 0.000, η^2^ = 0.130] and analytical thinking [*F*_(1, 111)_ = 4.844, *p* = 0.030, η^2^ = 0.042]. The interaction between misinformation accuracy judgment ratings and the level of support for Russia was insignificant [*F*_(1, 111)_ = 0.233, *p* = 0.336, η^2^ = 0.008]. These results show that prior attitudes are related to misinformation accuracy ratings to a greater extent than one's analytical thinking. Nevertheless, they support our second prediction that higher levels of analytical thinking will be negatively related to misinformation accuracy judgment.

To understand the direction of the interaction effects and to test our other predictions, we calculated several hierarchical multiple regressions. First, we tested how analytical thinking, attitude, and elicited emotions predicted the overall misinformation accuracy judgments while controlling for demographic variables (the residuals were not correlated [Durbin-Watson = 2.214], and no VIF value exceeded 1.7) (see Table 1). Of the demographic variables, only the respondent's level of education significantly predicted the misinformation accuracy judgment (ß = −0.24, *p* = 0.022); sex (0 = male, 1 = female; ß = −0.77, *p* = 0.409) and age (ß = 0.65, *p* = 0.534) were not significant predictors. In addition to demographic variables, the misinformation accuracy judgment was negatively related to the level of analytical thinking (ß = −0.21, *p* = 0.037) but was not related to other predictors in the model. Therefore, only analytical thinking was negatively related to overall misinformation accuracy judgment, as per our prediction (even when relevant sociodemographic variables were considered).

**Table 1:** Summary of hierarchical regression analysis for variables predicting misinformation accuracy judgments (*N* = 115).

**Variable**	***B***	***SE B***	**β**	***t***	***p***	***R^2^***	** $R_{\text{Adjusted}}^{2}$ **	**Δ*R^2^***
**Model 1**						0.06	0.03	0.06
Constant	3.02	0.22		13.87	0.00			
Age	0.00	0.00	0.07	0.62	0.53			
Gender^a^	−0.10	0.12	−0.08	−0.83	0.41			
Education level	−0.20	0.09	−0.24	−2.33	0.02^*^			
**Model 2**						0.15	0.08	0.09
Constant	2.62	0.45		5.79	0.00			
Age	0.00	0.00	−0.02	−0.14	0.89			
Gender^a^	−0.18	0.12	−0.14	−1.49	0.14			
Education level	−0.17	0.09	−0.20	−1.90	0.06			
Support for Ukraine	0.01	0.04	0.03	0.32	0.75			
Support for Russia	0.08	0.06	0.12	1.30	0.20			
Analytical thinking	−0.05	0.03	−0.21	−2.11	0.04^*^			
Positive emotions elicited by stimuli	0.24	0.19	0.12	1.23	0.22			
Negative emotions elicited by stimuli	0.07	0.06	0.11	1.18	0.24			

^*^p < 0.05. ^a^0 = male, 1 = female.

Next, we conducted separate hierarchical multiple regression analyses to test further relationships among the predictor variables and misinformation accuracy judgments.

For pro-Ukraine misinformation [the residuals were not correlated (Durbin-Watson = 2.154), no VIF value exceeded 1.7], of the demographic variables, only age (ß = 0.24, *p* = 0.024) was a significant predictor of the pro-Ukraine misinformation accuracy judgment. In contrast, gender (ß = −0.13, *p* = 0.153) and level of education (ß = −0.18, *p* = 0.081) did not have a significant relation with the pro-Ukraine misinformation accuracy judgment (see Table 2). Beyond demographic variables, the pro-Ukraine misinformation accuracy judgment was negatively related to the level of analytical thinking (ß = −0.23, *p* = 0.020), positively related to the level of support for Ukraine (ß = 0.19, *p* = 0.042), and positively related to positive emotions elicited by the stimuli (ß = 0.19, *p* = 0.040).

**Table 2:** Summary of hierarchical regression analysis for variables predicting pro-ukraine misinformation accuracy judgments (*N* = 115).

**Variable**	***B***	***SE B***	**β**	***t***	***p***	***R^2^***	** $R_{\text{Adjusted}}^{2}$ **	**Δ*R^2^***
**Model 1**						0.07	0.05	0.07^*^
Constant	3.35	0.28		11.94	0.00			
Age	0.01	0.00	0.24	2.29	0.02^*^			
Gender^a^	−0.22	0.15	−0.13	−1.44	0.15			
Education level	−0.20	0.11	−0.18	−1.76	0.08			
**Model 2**						0.24	0.18	0.17^**^
Constant	2.43	0.49		5.01	0.00			
Age	0.01	0.01	0.18	1.66	0.10			
Gender^*a*^	−0.33	0.15	−0.20	−2.18	0.03^*^			
Education level	−0.18	0.11	−0.17	−1.69	0.09			
Support for Ukraine	0.09	0.05	0.19	2.06	0.04^*^			
Support for Russia	0.02	0.08	0.03	0.28	0.78			
Analytical thinking	−0.08	0.03	−0.22	−2.36	0.02^*^			
Positive emotions elicited by stimuli	0.31	0.15	0.19	2.08	0.04^*^			
Negative emotions elicited by stimuli	0.09	0.08	0.10	1.15	0.25			

^*^p < 0.05. ^a^0 = male, 1 = female.

For pro-Russia misinformation (the residuals were not correlated [Durbin-Watson = 2.121], no VIF value exceeded 1.6), none of the variables included in the model were significant predictors of the pro-Russia misinformation accuracy judgment (see Table 3).

**Table 3:** Summary of hierarchical regression analysis for variables predicting pro-russia misinformation accuracy judgments (*N* = 115).

**Variable**	***B***	***SE B***	**β**	***t***	***p***	***R^2^***	** $R_{\text{Adjusted}}^{2}$ **	**Δ*R^2^***
**Model 1**						*0.08*	0.06	0.08^*^
Constant	2.69	0.27		9.90	0.00			
Age	−0.01	0.01	−0.14	−1.36	0.18			
Gender^a^	0.02	0.15	0.01	0.16	0.88			
Education level	−0.21	0.11	−0.20	−1.91	0.06			
**Model 2**						0.19	0.13	0.11^*^
Constant	2.46	0.60		4.08	0.00			
Age	−0.01	0.01	−0.20	−1.79	0.08			
Gender^a^	0.00	0.15	0.00	−0.01	0.99			
Education level	−0.13	0.11	−0.13	−1.25	0.21			
Support for Ukraine	−0.06	0.05	−0.12	−1.29	0.20			
Support for Russia	0.13	0.08	0.16	1.68	0.10			
Analytical thinking	−0.03	0.03	−0.09	−0.94	0.35			
Positive emotions elicited by stimuli	0.48	0.27	0.18	1.82	0.07			
Negative emotions elicited by stimuli	−0.01	0.06	−0.02	−0.21	0.83			

^*^p < 0.05. ^a^0 = male, 1 = female.

Finally, we tested the relationships among analytical thinking, prior attitude, elicited emotions, and misinformation accuracy judgment as predictors and the likelihood of engagement with content as an outcome while controlling for demographic variables (the residuals were not correlated [Durbin-Watson = 2.214], and no VIF value exceeded 1.7). Beyond demographic variables age (ß = −0.30, *p* = 0.004), gender (ß = −0.19, *p* = 0.035), and level of education (ß = −0.20, *p* = 0.057), the misinformation accuracy judgment (ß = 0.20, *p* = 0.033) and positive emotions elicited by stimuli (ß = 0.19, *p* = 0.043) were statistically significantly related to the likelihood of engagement with misinformation (see Table 4). When tested separately for the likelihood of engagement with pro-Ukraine and pro-Russia misinformation, none of the variables emerged as significant predictors. Therefore, misinformation accuracy judgment and positive emotions elicited by the stimuli were positively related to the reported likelihood to engage with misinformation after controlling for demographic variables, but analytical thinking did not have the expected negative relationship. Thus, our third prediction was rejected.

**Table 4:** Summary of hierarchical regression analysis for variables predicting the possibility of engagement (*N* = 115).

**Variable**	***B***	***SE B***	**β**	***t***	***p***	***R^2^***	** $R_{\text{Adjusted}}^{2}$ **	**Δ*R^2^***
**Model 1**						0.12	0.09	0.12^*^
Constant	1.49	0.20		7.52	0.00			
Age	0.01	0.00	0.30	2.98	0.00			
Gender^*a*^	−0.23	0.11	−0.19	−2.13	0.04^*^			
Education level	−0.15	0.08	−0.19	−1.93	0.06			
**Model 2**						0.19	0.07	0.07
Constant	0.89	0.42		2.13	0.04			
Age	0.01	0.00	0.32	2.85	0.01^**^			
Gender^*a*^	−0.24	0.11	−0.20	−2.16	0.03^*^			
Education level	−0.14	0.08	−0.17	−1.69	0.09			
Support for Ukraine	−0.01	0.03	−0.04	−0.38	0.70			
Support for Russia	0.01	0.06	0.01	0.12	0.91			
Analytical thinking	−0.02	0.02	−0.07	−0.72	0.47			
Positive emotions elicited by stimuli	0.41	0.18	0.21	2.28	0.02^*^			
Negative emotions elicited by stimuli	0.06	0.06	0.10	1.05	0.30			
Misinformation Accuracy Judgments	0.19	0.09	0.20	2.16	0.03^*^			

^*^p < 0.05. ^**^p < 0.01; ^a^0 = male, 1 = female.

## 5. Discussion

The key goal of this study was to explore the relationships among attitudes, analytical thinking, and the emotional perception of misinformation when evaluating its accuracy for congruent and incongruent content. Our first prediction was that there would be significant differences between misinformation accuracy judgments for pro-Ukraine information and those for pro-Russia information. The results showed significant differences that support biased evaluations of information. Pro-Russia misinformation was generally regarded by the participants as inaccurate, while pro-Ukraine misinformation resulted in more lenient accuracy judgments. These results signify a biased perception of information and show the importance of one's attitudes, resulting in myside bias, where information incongruent with one's prior beliefs is disregarded while congruent information is more readily accepted without additional scrutiny. As the stimuli were presented as Facebook posts by anonymized authors (without any information about the source or level of engagement, e.g., likes and shares), the central role was played by the participants' attitudes and trust toward congruent misinformation.

In previous research, analytical thinking has been identified as a predictor of a higher ability to identify a wide range of misinformation and reduced engagement. We were especially curious whether this would remain true when the topic was directly connected to the war happening in Ukraine and when the information was presented in a way that mimicked user-created content on Facebook rather than as news headlines. In our sample, analytical thinking had a statistically significant yet small effect on overall and pro-Ukraine misinformation accuracy judgments (higher analytical thinking reduced misinformation accuracy judgments for both kinds of misinformation). However, analytical thinking did not predict the evaluation of pro-Russia misinformation and had no relationship with the likelihood of engagement with the content. These findings suggest that when it comes to pro-Russia misinformation, a relatively fast and intuitive response (disregarding it as false) is more viable than an analytical approach. Overall, these findings are more consistent with the integrated model rather than the classical reasoning or motivated reasoning models individually; the participants showed strong evidence of myside bias, and their analytical thinking showed a relatively weak relation to accuracy judgments. Nevertheless, the results partly confirm our prediction that higher analytical thinking reduces belief in congruent misinformation. However, they also signify the role of prior attitudes and beliefs in the process.

As for demographic variables, education level showed a significant correlation with misinformation accuracy judgment—individuals with a higher level of education rated misinformation as less accurate. The participant's age was positively related to pro-Ukraine misinformation accuracy judgment. Overall, these results are not surprising, as the level of education can be associated with better analytical skills and higher overall skepticism and age with a more partisan viewpoint.

Regarding affective response, negative emotions elicited by the stimuli did not show any relationship with misinformation accuracy judgments, but positive emotions were related to higher levels of pro-Ukraine misinformation accuracy judgments. This might be explained by overall informational fatigue, where negative information surrounding the war topic is quickly disposed of or ignored. At the same time, positive misinformation focused on mythmaking and moral boosting is welcomed as a remedy for the grim reality.

Finally, participants indicated a higher likelihood of engaging with misinformation if they thought it was accurate and elicited positive emotions; however, the predicted negative correlations with analytical thinking were not found. From a demographic perspective, intent to engage was related to gender (lower for women), age (older individuals showed lower levels of engagement), and level of education (individuals with a higher education level were less likely to engage with misinformation).

The current study has a few limitations. The first is the small sample size and sampling biases that could have occurred. The effects of these limitations were minimized by conducting a preliminary power analysis and recruiting participants from a wide array of discussion groups. Second, as the study focused on participants supporting Ukraine, the variance of this support was low. Though it was something we anticipated and is adequate for this investigation, future studies could benefit from a more diverse sample. Third, the study's methodology could have influenced the results; although the misinformation posts were similar to those found on Facebook, they were not inserted in their natural environment, influencing the ecological validity of our study. However, this approach was justified as it increased the study's internal validity. Finally, while this study is only one piece of the puzzle that is trying to understand the subject, its findings can provide helpful insights or possibly motivate more research, which would be needed to make generalized conclusions.

In conclusion, our study illustrates the impact of prior beliefs on the evaluation of information. Though analytical thinking helps mitigate one's acceptance of misinformation, such (often unconscious) biases as myside bias ensure that it is mainly information that supports our beliefs that is valued as credible and trustworthy. Thus, analytical thinking should be deployed as a style of reasoning rather than a skill used selectively when it works in one's interests and is used to confirm one's perceptions. Future efforts in research and education addressing media literacy and analytical thinking should be adjusted to ensure one's ability to think analytically in less selective ways when one holds strong attitudes toward the topic at hand.

## Data availability statement

The raw data supporting the conclusions of this article will be made available by the authors, without undue reservation.

## Ethics statement

The studies involving humans were approved by the University of Latvia Ethics Committee for Research in Humanities and Social Sciences. The studies were conducted in accordance with the local legislation and institutional requirements. The participants provided their written informed consent to participate in this study.

## Author contributions

MP took the lead in planning, conducting the study, and writing the manuscript. All authors provided critical feedback and helped shape the research, analysis, contributed to the manuscript revision, read, and approved the submitted version.
